# Moisture-Dependent Strength Properties of Thermally-Modified *Fraxinus excelsior* Wood in Compression

**DOI:** 10.3390/ma13071647

**Published:** 2020-04-02

**Authors:** Edward Roszyk, Elżbieta Stachowska, Jerzy Majka, Przemysław Mania, Magdalena Broda

**Affiliations:** Department of Wood Science and Thermal Techniques, Faculty of Wood Technology, Poznań University of Life Sciences, Wojska Polskiego 38/42, 60-637 Poznań, Poland; edward.roszyk@up.poznan.pl (E.R.); ell.borow@gmail.com (E.S.); jerzy.majka@mail.up.poznan.pl (J.M.); przemyslaw.mania@up.poznan.pl (P.M.)

**Keywords:** compressive strength, ash, wood thermal modification, moisture content, mechanical properties

## Abstract

European ash (*Fraxinus excelsior* L.) is one of the species commonly used for wood thermal modification that improves its performance. The presented research aimed to investigate a moisture-dependent strength anisotropy of thermally-modified European ash in compression. Wood samples were modified at 180 °C and 200 °C. Their mechanical parameters were determined in the principal anatomical directions under dry (moisture content of 3%) and wet (moisture content above fibre saturation point) conditions. Effect of heat treatment temperature and moisture content on the ash wood mechanical parameters concerning each anatomical direction were determined. The results show that thermal treatment kept the intrinsic anisotropy of wood mechanical properties. It decreased wood hygroscopicity, which resulted in improved strength and elasticity measured for wet wood when compared to untreated and treated samples. Higher treatment temperature (200 °C) increased wood elasticity in compression in all the anatomical directions despite wood moisture content during the measurements. Multivariate analysis revealed that the modification temperature significantly affected the modulus of elasticity perpendicular to the grain, while in the case of compression strength, the statistically significant effect was observed only parallel to the grain. The results obtained can be useful from an industrial perspective and can serve as part of a database for further modelling purposes.

## 1. Introduction

Favoured by the increased pressure on the replacement of biocides with more environmentally friendly preservatives, thermal modification has been developed to increase wood biological durability and dimensional stability, and reduce its hygroscopicity. It is commercially by far the most advanced wood modification technology in the market with the highest economic importance. Numerous production plants worldwide utilise heat treatment techniques carrying out modification processes in open and closed systems that differ mainly in the applied treatment temperature (always above 150 °C) and the medium used to exclude oxygen [1,2,3].

Thermal modification results in chemical changes in wood cell wall polymers. An organic-acid mediated process causes decomposition of hemicelluloses. It also leads to some lignin cleavage, which results in its depolymerisation in a first stage, and then to auto condensation through the formation of methylene bridges connecting aromatic rings. The crystallinity of cellulose increases and structural changes in amorphous cellulose can also be observed. However, the extent of chemical reactions is rather moderate, their effect in the form of an increased cross-linking of cell wall polymers results in the reduction of wood hygroscopicity and contributes to the new properties of thermally-modified wood: an improved dimensional stability and wood decay resistance as well as its darker colouration [1,3,4,5,6,7,8,9]. Thus, the method enables the use of low durability local wood outdoors or for applications that require excellent dimensional stability [1,10,11,12,13]. It was also showed that thermal treatment improves the acoustic parameters of wood [14]. Unfortunately, the changes in the chemical composition and structure of the cell wall polymers and the resulting anatomical alterations and lower density of wood affect its mechanical behaviour. While the surface hardness of thermally-treated wood is improving, its other mechanical properties, such as compression, shear and bending strength or stiffness are considerably deteriorating according to the treatment conditions, which limits its use in structural applications [1,2,3,9,15,16,17,18].

European ash (*Fraxinus excelsior* L.) is one of the species commonly used for wood thermal modification. The treatment significantly improves its performance making it suitable for different uses. In respect to biological effectiveness, studies have shown that heat treatment considerably enhances ash resistance to brown- and white-rot fungi and soil-inhabiting microorganisms (for heat treatment at a temperature >200 °C) [19]. However, it is not effective against thermites [19,20]. Thermal modification reduces the equilibrium moisture content (EMC) of ash [3,21,22] and decreases its water retention value [23], enhancing its dimensional stability [22,24,25]. It also alters the natural colour of wood, making it darker with the increasing severity of the treatment [3,21,26] as well as improves its resistance to weathering factors as regards colour stability, surface quality, and the reduction rate for strength properties [21,27,28]. The resulting improved performance of thermally-modified ash makes it an excellent and popular raw material for dump premises and outdoor applications, including terrace boards, facades, claddings, garden furniture, and for indoor use requiring high dimensional accuracy, such as floorboards, stairs, or panelling boards.

The broad applicability of heat-treated ash prompted researchers to study the influence of treatment conditions on its particular mechanical properties. Pleschberger et al. [26] studied the fracture behaviour of ash modified at a temperature of 200, 210, and 220 °C in radial/longitudinal and tangential/longitudinal direction at 65% air relative humidity (RH). Their results revealed that the specific fracture energy and the maximum breaking load decreased with increasing intensity of the applied treatment. The fracture toughness of ash wood modified at a temperature of 180, 200, and 230 °C was also determined by Majano-Majano et al. [3] at 33, 65, and 95% RH, respectively. The obtained lowered values of the critical stress intensity factor indicate that crack initiation in heat-treated wood becomes easier and the crack propagation phase requires less energy and takes place in a more brittle manner with increasing severity of the treatment. The observed increased brittleness of the thermally-modified ash eliminates it from structural applications. Standfest and Zimmer [29] investigated the influence of three different treatment temperatures (160, 180, and 200 °C) on the Brinell hardness of ash in a longitudinal, radial, and tangential direction. The results obtained showed that the two lower temperatures applied did not affect wood hardness in radial and tangential directions, while the heat treatment at a temperature of 200 °C reduced it. Unexpectedly, the thermal treatment at all the three temperatures applied increased the hardness values in a longitudinal direction [29].

In contrast, the study by [24] showed a reduction of ash hardness in the principal anatomical directions after heat treatment at a temperature of 200 °C. Hannouz et al. [16] carried out a wide range of mechanical characteristics (bending, tension parallel, and perpendicular to the grain, compression parallel, and perpendicular to the grain and shear) at 65% RH on ash wood thermally-treated at 210 °C. They found out that heat treatment decreases strength properties of ash except for compression parallel to the grain, and increases its modulus of elasticity perpendicular to the grain. On the other hand, the results of mechanical tests on ash modified at 200 °C performed by Govorcin et al. [24] showed the decrease of modulus of rupture (MOR) and compression strength in the longitudinal direction (the moisture content (MC) of wood samples was different than in the case of Hannouz (4% and 12%, respectively).

The thermal modification does not affect all the ash wood mechanical properties in the same way and to the same extent. Therefore, to enable the broader use of heat-treated ash, accurate and comprehensive knowledge about its mechanical behaviour under various conditions is necessary.

Our previous research on the mechanical parameters of wood modified at 190 and 200 °C with a moisture content of 4% and 12% subjected to compression in radial and tangential directions revealed that the changes in wood mechanical behaviour depend on the modification temperature and wood moisture content [30,31]. Compressive strength in a radial direction for heat-treated ash decreased much more than for untreated control with the increasing wood moisture content. The applied treatment significantly deteriorated wood compression strength except for wood samples of 4% MC modified at 190 °C, in which the mechanical parameters improved [30]. In the case of the mechanical parameters measured in a tangential direction, a similar trend has been observed. However, samples with 4% MC modified at 190 °C showed the conventional specific strength similar to that of the control wood. For those modified at 200 °C, the linear elasticity modulus was comparable with untreated wood, while the other measured parameters were lower. The observed minor effect of wood moisture content on the elastic energy of the heat-treated wood indicates the permanent and irreversible increase in its brittleness [31].

Studies on creep of thermally-modified ash subjected to compression in tangential and radial directions and simultaneously wetted from 6% MC to above the fibre saturation point (FSP), revealed that thermal modification reduces the strain of ash subjected to compression perpendicular to the grain to a degree proportional to the mass loss. Moreover, although upon the thermal treatment, the mass loss of wood took place, at the MC of 6% practically the same modulus of elasticity (MOE) and compressive strength (R_c_) as for unmodified wood was observed. After wetting to MC higher than the FSP, the wood modified at 200 °C showed significantly higher MOE and R_c_ than the wood modified at 180 °C and untreated wood. Resulted from thermal modification, the reduction in wood hygroscopicity reduced the range of changes in mechanical properties of wood caused by the increase in its MC to the FSP [32].

The presented research aims to contribute to a better understanding of a moisture-dependent strength anisotropy of thermally-modified European ash (*Fraxinus excelsior* L.) in compression. The mechanical properties of wood were determined in the principal anatomical directions under the conditions similar to the conditions of its use, i.e., at dry (MC of 3%) and wet state (MC > 30%). Effect of heat treatment temperature and moisture content on the mechanical parameters of ash concerning each anatomical direction were determined, which can be useful from an industrial perspective, and together with the results of previous studies [3,16,24,26,29,30,31,32], provides a set of results useful for further modelling purposes.

## 2. Materials and Methods

### 2.1. Thermal Modification and Wood Sampling

**Figure 1:**
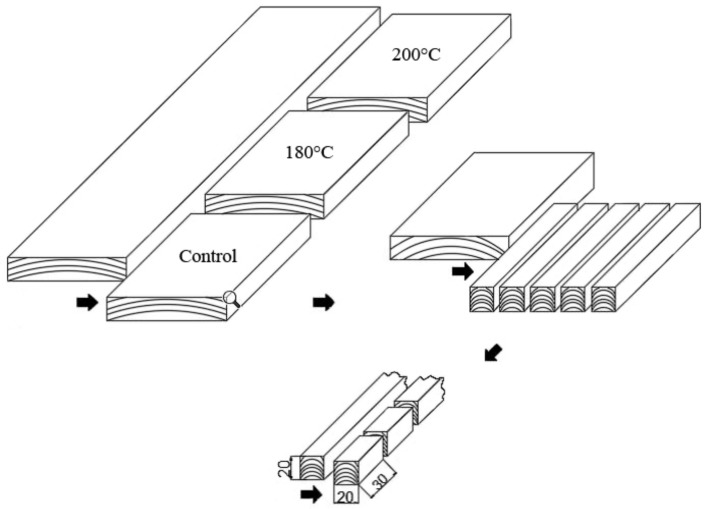
The sample preparation scheme.

Five kiln-dried boards of *Fraxinus excelsior* L. wood (raw dimensions of 25 × 150 × 1000 mm for the thickness, width, and length, respectively) were cut into three samples according to the scheme (Figure 1). One of the samples was used as a control, and the other two were thermally modified at 180 and 200 °C, respectively. The thermal modification was conducted following the ThermoWood method [33]. It included the following phases: an initial step in moist air followed by heating in superheated steam after the temperature reached 130 °C, a maximum heating phase (T = 180 °C or 200 °C, t = 3 h), and a cooling stage with superheated stem followed by cooling in moist air only.

Control and modified samples were cut into slats with cross-sectional dimensions of 20 × 20 mm. Then final specimens were prepared with the dimensions of 20 × 20 × 30 mm (tangential × radial × longitudinal direction), as prescribed by ISO 13061-17:2017 [34] and ISO/FDIS 13061-5 [35] standards for determination of strength in compression parallel to the grain and perpendicular to the grain, respectively.

The obtained samples were diverse in tree-ring widths to simulate natural heterogeneity of wooden material used in the industry. Wood density measured at 8% MC using a stereometric method was between 526 and 673 kg × m^−3^ (mean value was 596 kg × m^−3^).

### 2.2. Conditioning of the Specimens

Wood specimens were divided into two groups to test the mechanical properties of thermally-modified wood under the conditions similar to the conditions of its use. One of them was moisture-conditioned to achieve MC of 3% (dry conditions), and the second has MC above the fibre saturation point (FSP) (wet conditions).

To obtain 3% MC, the control wood specimens and those modified at a temperature of 180 °C were placed in a desiccator containing a saturated aqueous solution of lithium chloride and potassium acetate, respectively. Wood specimens treated at a temperature of 200 °C were seasoned under ambient laboratory conditions (T = 20 ± 1 °C, air relative humidity = 40 ± 5%) until a constant mass was achieved. 

To obtain MC > 30% (above FSP), all the specimens were first pre-seasoned in a desiccator above the water surface (T = 20 ± 1 °C) to prevent their cracking and then they were immersed in water until a constant mass was achieved.

### 2.3. Compression Tests

Before the compression tests, the mass of the conditioned specimens and their dimensions in the principal anatomical directions were measured using an analytical balance accurate to 0.001 g (Sartorius GmbH, Göttingen, Germany) and a digital calliper with accuracy to 0.01 mm, respectively, to calculate wood density (according to ISO 13061-2:2014).

Compression tests in all three anatomical directions were conducted using a numerically controlled test machine Zwick Z050TH (Zwick/Roell, Ulm, Germany). Ten samples of each variant (two modification modes and untreated control at two different MC) were tested in each direction. The modulus of elasticity (MOE) and stress at proportionality limit (so-called compressive strength perpendicular to the grain or relative strength (R_cT_ and R_cR_ in a tangential and radial direction, respectively) were determined. Additionally, stress to failure (so-called compressive strength—R_cL_) was determined for a longitudinal direction.

### 2.4. Statistical Analysis

The experimental data were statistically analysed using STATISTICA 13.3 software (TIBCO Software Inc., Palo Alto, CA, USA). A multivariate analysis of variance (ANOVA) was performed to determine whether moisture content, the temperature of modification and anatomical direction affected mechanical parameters of examined wood. Significance was established at the *p* < 0.05. Tukey’s honest significance test was applied to find means that are significantly different from each other.

## 3. Results and Discussion

Thermal treatment of ash resulted in a decrease in wood density (measured at 3% MC) from 591 ± 53 kg × m^−3^ to 579 ± 51 and 563 ± 45 kg × m^−3^ for wood modified at 180 and 200 °C, respectively, with a concomitant mass loss of about 1.8% for 180 °C and 6% for 200 °C. Resulting from the degradation of cellulose and hemicelluloses, the observed reduction in wood mass and density increases with the increase of the modification temperature and is typical for thermal treatment, and is in line with the results obtained by others [3,16,31,32,36].

The results of the elastic and strength properties under compression load measured in the three anatomical directions for untreated and treated ash, together with the results of post-hoc Tukey’s test, are presented in Table 1.

The values of MOE and R_c_ obtained in compression tests (Table 1) demonstrate clearly the orthotropic nature of wood, due to which its mechanical properties in the three anatomical directions are unique and independent [37]. For untreated ash with a density of about 591 kg × m^−3^ and 3% MC, MOE was 844, 1356, and 11265 MPa in the tangential (T), radial (R), and longitudinal (L) direction, respectively, showing anisotropy and a typical order MOE_L_ >> MOE_R_ > MOE_T_ [38]. The corresponding R_c_ values were 8.1, 6.3, and 79.9 MPa in the T, R, and L direction, respectively. A similar relation between MOE and R_c_ values in different anatomical directions measured for wood differing in the treatment applied and MC was found. For comparison, the MOE values for ash with a density of 670 kg × m^−3^ and 11% MC was 800, 1510, and 13,700 MPa in the T, R, and L direction, respectively [39].

The results of a multivariate analysis of variance (ANOVA) are presented in Table 2 and Table 3. They show statistical significance of the relationships between particular variables in the experiments performed in the study, such as a temperature of modification, anatomical direction and moisture content, and the mechanical parameters measured for thermally-modified wood. 

Besides the apparent influence of moisture content and the anatomical direction on MOE, the results indicate a significance (*p* < 0.05) of a modification temperature on this parameter only perpendicular to the grain (Table 2). The interactions between particular factors can be considered non-significant, excluding the combined effect of moisture content and the anatomical direction (a × c).

With regard to R_c_, the results of the variance analysis indicate the significant effect of the modification temperature on this parameter only parallel to the grain (Table 3). For both tangential directions (perpendicular to the grain), the significant interactions between all the examined factors exist.

**Figure 2:**
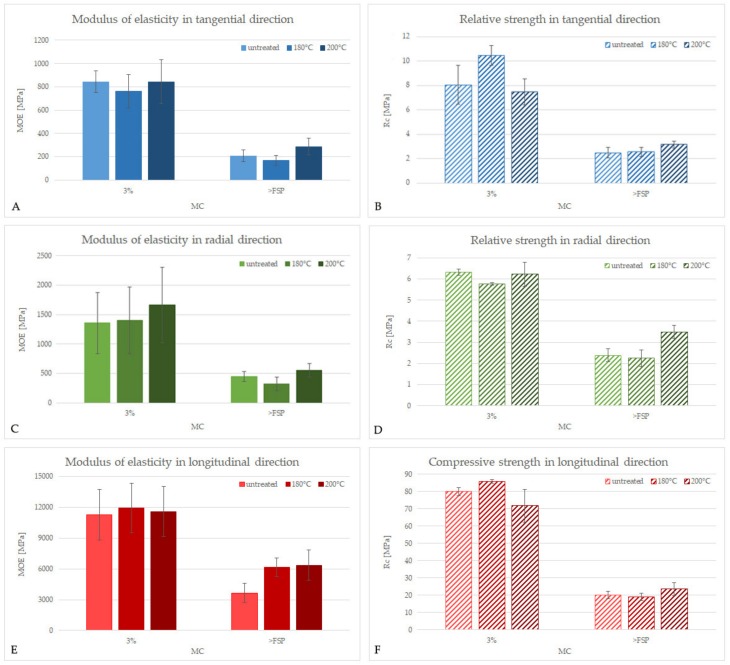
Modulus of elasticity (MOE) and relative/compressive strength (R_c_) for unmodified and thermally-modified ash measured in the tangential (**A**,**B**), radial (**C**,**D**), and longitudinal direction (**E**,**F**).

Irrespective of the treatment applied and the anatomical direction, modulus of elasticity and relative/compressive strength of ash revealed typical high dependency on wood moisture content (Table 1, Figure 2), decreasing significantly with an increase of MC due to the softening effect of water on the cellulose microfibrils in the wood cell walls. For untreated wood, MOE was reduced from 844 to 207 MPa in T, from 1356 to 446 MPa in R, and from 11265 to 3643 MPa in longitudinal direction L with an increase of MC from 3% to above the fibre saturation point (>30%). R_c_ decreased from 8.1 to 2.5 MPa in T, from 6.3 to 2.4 MPa in R, and from 79.9 to 19.9 MPa in L, respectively. Similar relationships were also observed for heat-treated ash (Figure 2). The results of static bending measurements and dynamic tests performed on ash wood by Niemz et al. [39] revealed a dependency of MOE in all anatomical direction on the MC as well.

**Figure 3:**
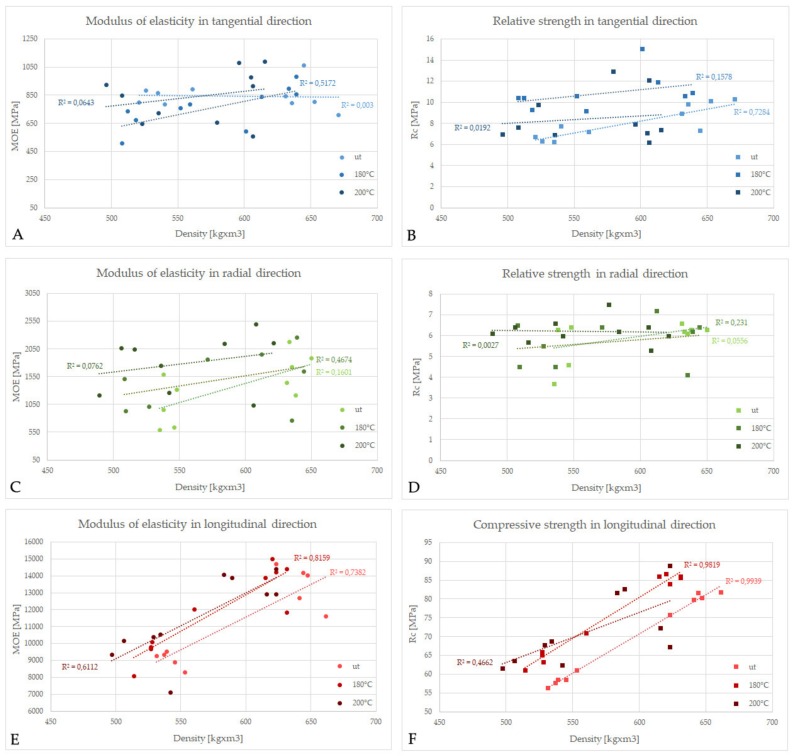
Modulus of elasticity (MOE) and relative/compressive strength (R_c_) for a different density of unmodified and heat-treated ash measured at 3% MC in the tangential (**A**,**B**), radial (**C**,**D**), and longitudinal direction (**E**,**F**).

When analysing the data obtained (Table 1), it is clear that in the case of wood at 3% MC, the thermal modification applied did not significantly change wood mechanical properties despite the apparent alteration in its chemical composition visible in the mass/density loss of wood observed after heat treatment. The apparent increase in the mean values of R_cT_ and R_cL_ after treatment at 180 °C is an effect of huge dispersion of the data obtained (Figure 3) resulted from the specific selection of research material dedicated for these experiments (wood with naturally diversified density between 526 and 673 kg × m^−3^) since the aim was to characterise the properties of ordinary heat-treated wood for industrial purposes.

In the case of wet wood (>FSP), however, although heat-treatment at 180 °C did not markedly affect the wood mechanical parameters except MOE in the longitudinal direction, the effect of modification at 200 °C was substantial. MOE increased from 207 to 287 MPa in T, from 446 to 551 MPa in R, and from 3643 to 6342 MPa in L, and R_c_ increased from 2.48 to 3.17 MPa in T, from 2.39 to 3.49 MPa in R, and from 19.9 to 23.3 MPa in L, respectively.

The positive effect of thermal treatment on the reduction of wood hygroscopic character has already been known [40]. The analysis of isotherms within the Hailwood–Horrobin model has shown that the changes take place in the range of chemisorption and in that of capillary sorption. The moisture content of wood treated at 190 and 210 °C may decrease by half. Moliński et al. [41] thermally modifying ash wood, stated that the fibre saturation point in wood decreased from 28% to 15%. It is well known that the mechanical parameters of wood depend on its moisture content, but this effect can only be observed in the hygroscopic range, where free water does not affect the strength of the wood.

In the presented study, the mechanical parameters of wood after modification were compared with the reference data (control wood—unmodified) after conditioning the samples under similar conditions of relative humidity and temperature. In other words, the mechanical parameters of modified wood were determined at a lower moisture content of the wood tissue than in the case of control samples (see Table 1). The increased values of MOE and R_c_ can be explained by the combined effect of chemical and structural changes in wood polymers that occur during treatment at 200 °C. On the one hand, degradation of hemicelluloses, an increase in cellulose crystallinity, and the polycondensation of lignin upon heat-treatment decrease the number of hydroxyl groups available for reaction with water molecules, which results in the limited equilibrium moisture content, thus making wood less pliable [4,6,42]. The increase of the amount of crystalline cellulose and in cross-linking of the lignin network enhance the rigidity of microfibrils and the structure around them, additionally contributing to increase in MOE and compressive strength [43]. It is worth noting that the effect of lignin polycondensation, as well as an increase in cellulose crystallinity, are the most prominent in the longitudinal direction (MOE almost doubled when comparing untreated wood and heat-treated at 200 °C), which is attributed to the molecular structure of wood and the cell wall, and anisotropy of microfibrils [4,43].

The results obtained show that thermal treatment alters the natural anisotropy of wood mechanical parameters in all the anatomical directions, but the effect depends on wood moisture content (Table 4).

Generally, for control samples, the E_L_/E_T_ ratio has the highest value for both humidity levels. The lowest is the value of E_T_/E_R_ ratio, which is fully understood taking into account that in both transverse directions the modulus of elasticity is comparable (although usually higher in the radial direction than in the tangential) and definitely lower than along the fibres. In the case of modified samples, these relationships remain the same, but the proportions between particular elastic ratios changed.

There is a visible difference in the effect of modification temperature on elastic ratios between dry and wet samples. For dry samples, thermal modification at 180 °C resulted in a slight increase of the E_L_/E_T_ ratio by 16.4% and a decrease in E_T_/E_R_ ratio by 12.5%, while modification at 200 °C did not affect the E_L_/E_T_, but decreased E_L_/E_R_ and E_T_/E_R_ by 16.9% and 18.5%, respectively. When analysing the elastic ratios for wet samples, it can be seen that heat treatment (at both temperatures) significantly increased all of them, whereby the highest increase was recorded for E_L_/E_R_ ratio (by 134.1% and 40.2% for 180 °C and 200 °C, respectively). The observed changes in elastic ratios for wet wood are the result of differences in wood MC during measurements. However, such an increase in the measured ratios confirms the suitability of modified wood for use in humid conditions, including outdoors.

**Figure 4:**
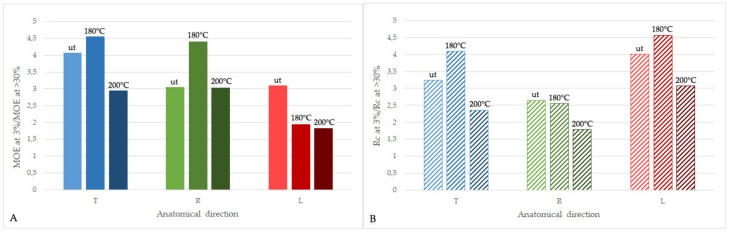
Anisotropy of the ratio between MOE (**A**) and R_c_ (**B**) values at MC of 3% and >30% for untreated (ut) and thermally-modified ash (at 180 and 200 °C) in the tangential (T), radial (R), and longitudinal (L) direction.

The differences in anisotropy of wood mechanical parameters in compression, induced by the applied heat treatment, resulted in variations in wood moisture content. It can be clearly seen when comparing the ratio between MOE (or R_c_) values at eMC of 3% and >30% for untreated and treated wood in different anatomical directions (Figure 4).

In the case of MOE values (Figure 4A), heat treatment had the lowest effect on the changes in elasticity of dry and wet wood measured in the longitudinal direction (MOE at 3% / MOE > 30% was about 1.8–1.9). In the case of transverse directions, however, there is a significant difference in the effect of temperature of treatment on wood elasticity in compression. For wood treated at 180 °C, the MOE ratios increased in comparison with untreated wood by about 0.5, and almost 1.5 in T and R, respectively, while higher treatment temperature (200 °C) caused a decrease in MOE ratio in T by about 1, but did not changed MOE ratio in R. These observations point out that for heat-treated wood, the changes in elastic properties in compression are highly dependent on wood moisture content when measured in the radial or tangential direction, but less prone to moisture changes when measured in the longitudinal direction. On the other hand, when comparing R_c_ ratios (Figure 4B), the smallest changes due to the moisture variations were observed in the radial direction, while the most significant were calculated in the longitudinal directions. As it is clear from Figure 3, there is a direct correlation between wood density and mechanical parameters in compression only in the case of the longitudinal direction, while in the transverse direction they are dependent also on other factors.

## 4. Conclusions

To conclude, the thermal treatment decreased hygroscopicity of ash wood, which resulted in improved MOE and R_c_ in compression measured for wet wood when comparing untreated and treated samples. Heat treatment kept intrinsic anisotropy of wood mechanical parameters in compression, maintaining a standard MOE order of MOE_L_ >> MOE_R_ > MOE_T_ and R_c_ order of R_cL_ >> R_cR_ < R_cT_. The increased moisture content of wood did not affect the order mentioned above. However, it altered proportions between particular measured values. Higher treatment temperature (200 °C) increased wood elasticity in compression in all the anatomical directions despite wood moisture content during the measurements. Based on the analysis of variance, it can be stated that the modification temperature significantly affected the modulus of elasticity perpendicular to the grain. At the same time, in the case of compression strength, the statistically significant effect was observed only parallel to the grain. With respect to R_c_, the significant interactions between all the examined factors were found in both directions perpendicular to the grain. The results obtained suggest the possibility of applying thermally-modified ash wood in humid conditions.

## Figures and Tables

**Table 1:** Density, real moisture content (MC), modulus of elasticity (MOE) and relative/compressive strength (R_c_) for thermally-modified and unmodified (ut) ash equilibrated to achieve different moisture content (eMC) in the principal anatomical directions (T—tangential, R—radial, L—longitudinal).

eMC	Treatment Applied	Density (kg × m^−3^)	MC (%)	MOE_T_ (MPa)	R_cT_ (MPa)	MOE_R_ (MPa)	R_cR_ (MPa)	MOE_L_ (MPa)	R_cL_ (MPa)
3%	ut	591 ± 53	3.6 ± 0.1	844 ± 94 ^a^	8.1 ± 1.6 ^a^	1356 ± 521 ^a^	6.3 ± 0.2 ^a^	11265 ± 2473 ^a^	79.9 ± 2.4 ^a^
	180 °C	579 ± 51	2.7 ± 0.1	764 ± 143 ^a^	10.5 ± 0.8 ^b^	1405 ± 567 ^a^	5.8 ± 0.1 ^a^	11910 ± 2410 ^a^	85.7 ± 1.0^b^
	200 °C	563 ± 45	2.6 ± 0.1	844 ± 187 ^a^	7.5 ± 1.1 ^a^	1666 ± 638 ^a^	6.2 ± 0.6 ^a^	11589 ± 2421 ^a^	71.7 ± 9.5 ^a^
>FSP	ut	930 ± 64	97.1 ± 9.5	207 ± 50 ^a^	2.5 ± 0.4 ^a^	446 ± 88 ^a^	2.4 ± 0.3 ^a^	3643 ± 937 ^a^	19.9 ± 2.2 ^a^
	180 °C	903 ± 50	85.7 ± 4.0	168 ± 42 ^a^	2.6 ± 0.4 ^a^	319 ± 117 ^a^	2.3 ± 0.40 ^a^	6137 ± 902 ^b^	18.8 ± 2.0 ^a^
	200 °C	833 ± 44	68.7 ± 4.4	287 ± 70 ^b^	3.2 ± 0.3 ^b^	551 ± 112 ^b^	3.5 ± 0.3 ^b^	6342 ± 1470 ^b^	23.3 ± 3.8 ^b^

^a,b^ different superscripts denote a statistically significant (*p* < 0.05) difference between mean values (*n* = 10 for each set of samples) according to Tukey’s honest significant difference test.

**Table 2:** Analysis of variance taking into account the effect of moisture content, modification temperature and the anatomical direction on the modulus of elasticity (MOE) perpendicular and longitudinal to the grain for thermally-modified ash (*Fraxinus excelsior* L.) wood.

Direction	Effect	SS	df	MS	F	*p*-Value
Perpendicular to the grain	Intercept	42,869,394	1	42,869,394	400.2830	0.000
	Moisture content (a)	13,725,849	1	13,725,849	128.1619	0.000
	Temperature of modification (b)	629,926	1	629,926	5.8818	0.018
	Anatomical direction (c)	3,795,272	1	3,795,272	35.4375	0.000
	a × b	601	1	601	0.0056	0.940
	a × c	1,079,301	1	1,079,301	10.0777	0.002
	b × c	71,365	1	71,365	0.6664	0.417
	a × b × c	1443	1	1443	0.0135	0.908
	Error	7,175,547	67	107,098		
	Intercept	3.24 × 10^9^	1	3.24 × 10^9^	883.88	0.000
Longitudinal to the grain	Moisture content (a)	3.04 × 10^8^	1	3.04 × 10^8^	82.93	0.000
	Modification temperature (b)	3.31 × 10^4^	1	3.31 × 10^4^	0.01	0.925
	a × b	6.92 × 10^5^	1	6.92 × 10^5^	0.19	0.666
	Error	1.32 × 10^8^	36	3.66 × 10^6^		

SS—a sum of squares, df—degrees of freedom, MS—mean squares, F—Fisher’s F-test.

**Table 3:** Analysis of variance taking into account the effect of moisture content, modification temperature and the anatomical direction on relative/compressive strength (R_c_) perpendicular and longitudinal to the grain for thermally-modified ash (*Fraxinus excelsior* L.) wood.

Direction	Effect	SS	df	MS	F	*p*-Value
Perpendicular to the grain	Intercept	1986.241	1	1986.241	4358.716	0.000
	Moisture content (a)	398.813	1	398.813	875.178	0.000
	Temperature of modification (b)	0.639	1	0.639	1.402	0.241
	Anatomical direction (c)	42.039	1	42.039	92.253	0.000
	a × b	21.639	1	21.639	47.486	0.000
	a × c	40.585	1	40.585	89.061	0.000
	b × c	18.596	1	18.596	40.808	0.000
	a × b × c	9.477	1	9.477	20.796	0.000
	Error	30.532	67	0.456		
	Intercept	79,584.5	1	79,584.5	2506.564	0.000
Longitudinal to the grain	Moisture content (a)	26,597.4	1	26,597.4	837.702	0.000
	Modification temperature (b)	179.74	1	179.74	5.661	0.024
	a × b	691.92	1	691.92	21.792	0.000
	Error	984.26	31	31.75		

SS—a sum of squares, df—degrees of freedom, MS—mean squares, F—Fisher’s F-test.

**Table 4:** Elastic ratios (E) for thermally-modified and unmodified (ut) ash equilibrated to achieve different moisture content (eMC).

eMC	Treatment Applied	E_L_/E_T_	E_L_/E_R_	E_T_/E_R_
3%	Ut	13.4	8.3	0.622
	180 °C	15.6	8.5	0.544
	200 °C	13.7	6.9	0.507
>FSP	Ut	17.6	8.2	0.464
	180 °C	36.5	19.2	0.527
	200 °C	22.1	11.5	0.521
